# Drivers of global media attention and representations for antimicrobial resistance risk: an analysis of online English and Chinese news media data, 2015–2018

**DOI:** 10.1186/s13756-021-01015-5

**Published:** 2021-10-23

**Authors:** Qiuyan Liao, Jiehu Yuan, Meihong Dong, Pauline Paterson, Wendy Wing Tak Lam

**Affiliations:** 1 School of Public Health, Li Ka Shing Faculty of Medicine, The University of Hong Kong, 7 Sassoon Road, Pokfulam, Hong Kong China; 2 Department of Infectious Disease Epidemiology, London School of Hygiene and Tropical Medicine, London, UK

**Keywords:** Antimicrobial resistance, Risk communication, Risk representation

## Abstract

**Background:**

How antimicrobial resistance (AMR) risk is communicated in news media can shape public understanding and the engagement of different sectors with AMR. This study examined online news media attention for AMR risk and analyzed how AMR risk was communicated using a global sample of English and Chinese news articles.

**Methods:**

A total of 16,265 and 8335 English and Chinese news relevant to AMR risk, respectively, published in 2015–2018 were retrieved from a professional media-monitoring platform, to examine media attention for AMR and its drivers, of which, 788 articles from six main English-speaking countries and three main Chinese-speaking territories were drawn using constructed-week sampling for content analysis.

**Results:**

Media attention mainly fluctuated around official reports or scientific discovery of AMR risks or solutions but seldom around reports of inappropriate antimicrobial use (AMU), and not consistently increased in response to World Antimicrobial Awareness Week. The content analysis found that (1) heterogeneous medical terminologies and the ‘superbug’ frame were most commonly used to define AMR or AMR risk; (2) a temporal increase in communicating microbial evolution as a process of AMR was identified but communication about inappropriate AMU in general consumers as the cause of AMR remained inadequate; and (3) the multifaceted consequences of AMR and individual actions that can be taken to tackle AMR were inadequately communicated.

**Conclusions:**

The media should be encouraged or reoriented to communicate more about actions that can be taken by general consumers to enable collective actions and the multifaceted conseuqences of AMR to encourage one-health approach for tackling AMR.

## Introduction

Antimicrobial resistance (AMR) is one of the most important global health concerns, posing threat to human and animal health, food and environment security, and global economy [1]. AMR is driven by multifaceted causes involving for instance, inappropriate prescription of antibiotics in healthcare, overuse or misuse of antibiotics in consumers and animals as well as discharge of antibiotic residues to the ecological system, which cannot be addressed by any single sectors. This put forth the initiative of the One Health concept for tackling AMR [2]. One Health emphasizes the interdependencies between human, animal and environment health, and highlights the importance of intersectoral collaboration involving government, healthcare, agricultural, animal, food industry and educational sectors to address AMR [2]. News media coverage and how AMR risk is conveyed or framed in news media can powerfully shape public perceptions of AMR risk and the engagement of multiple sectors regarding the issue of AMR. It was found that in the UK, news articles from main national newspapers commonly represented AMR as a social problem that is mainly caused by ‘dirty’ hospitals or by ‘others’ who misuse/overuse antibiotics, while the role of other social actors (e.g. agricultural workers, consumers) was marginalized [3]. The US news increasingly framed AMR as a problem caused by antibiotic use in livestock production in recent years, but it was usually the poor animal welfare practices to be blamed rather than general consumers who drove these practices [4]. The analysis of news about AMR from television, print newspapers and digital sources from Australia in 2017 revealed that most of the news that year was about discovery of new scientific discoveries related to AMR or AMR solutions, which may put individuals at the position of ignorance, lacking agency for the AMR agenda [5]. These studies [3–5] focused on AMR news of different periods and used different methods to guide their analyses, making it difficult to compare the findings across the three countries. Nevertheless, overemphasizing one single sector’s responsibility in the issue of AMR may be ineffective for engaging multisectoral effort to tackle AMR. News articles tend to blame otherness for causing AMR risk [3, 4, 6, 7], which may somewhat undermine the public’s motivation for taking collective action to tackle AMR. AMR can also be characterized as a global crisis and catastrophe (e.g., “superbug crisis”) that evokes urgency of AMR problems but may also trigger feelings of helplessness, and thus paralyze individual actions [6, 7]. Moreover, news articles seldom mentioned actions that individuals could do to tackle AMR [3, 5]. Overall, current news reports on AMR may contribute to the public’s feeling of uncontrollability and low engagement of different sectors in tackling AMR.

Existing studies on news media representations of AMR mainly focused on English news media from high-income countries [3–8] but seldom in less-developed countries such as India, China and South Africa that consumed the largest amount of antibiotics in the world [9–11]. Despite a growing research interest in the epidemiology of AMR in low- and middle-income countries [12–14], the limited research in media representation of AMR risk in these countries may be due to a general overlook of the importance of media context in shaping public perception, policies and stakeholder engagement regarding the issue of AMR. This may drive primarily technological rather than multidisciplinary approaches to AMR in these countries. Most existing studies analysed news media data on AMR in one single country for a short period [8, 15], and mainly analysed printed media despite a global increase in the consumption of online news with the expansion of digital communication technologies [6, 16].

The aim of this study was to provide an analysis of news media representations of AMR risk, focusing on both English and Chinese online news articles published in 2015–2018 globally. English and Chinese are the two most popular languages spoken by the world’s population. Therefore, including both the English and Chinese news can provide a more comprehensive picture regarding the global representations of AMR risk in news media and enable comparative analysis across the two media contexts to inform how cultural, political, and other social contexts shaped media representations of AMR risk and subsequently solutions to AMR. News articles of other languages in which our research team were not competent were not included. We chose a timeframe of 5 years from 2015 to 2018 to enable analysis of the temporal trend in media attention to AMR risk and allow examination of how the One Health approach was implemented since it was urged in the World Health Assembly for the global action plans on AMR [17]. The specific objectives included: (1) To examine online news media attention for AMR risk and its temporal change from 2015 to 2018 and media attention for AMR risk during World Antimicrobial Awareness Week (WAAW) which has been set at every November since 2015; (2) To identify key events that drove media attention for AMR risk; (3) To conduct content analysis on how AMR risk was communicated in online news media.

## Methods

### Data retrieval

News articles were retrieved from the platform provided by Meltwater, a professional media-monitoring company that enables access to ~ 250,000 online newspapers globally [18] and has demonstrated to provide data of high quality for academic research [19]. English and Chinese online news published between January 2015 and December 2018 were retrieved using a list of key words in English and Chinese including general AMR terms (e.g., “antibiotic resistance”; “antimicrobial resistance”; and “superbugs”) and AMR terms specific to pathogens (e.g., “clostridium difficile”), drugs (e.g., “colistin”) and diseases [e.g., “Methicillin-resistant Staphylococcus Aureus” (MRSA)] (Appendix Table 5).

### News screening and sampling

The retrieved news articles published by the top 25% media agencies ranked by Reach (i.e., number of subscribers of a specific media agency) were included for eligibility screening. Two research assistants independently screened for the eligibility of the retrieved news based on the pre-set inclusion and exclusion criteria (Appendix Table 6). This generated a pool of eligible news articles for analysing media attention for AMR risk and the related key events. Subsequently, news articles from six main English-speaking countries—the United States (US), the United Kingdom (UK), Canada, Australia, India, South Africa, and three main Chinese-speaking territories—Mainland China, Hong Kong and Taiwan were sampled for in-depth content analysis, because 84.1% and 95.8% of all English and Chinese news articles, respectively, were from these countries and territories. Constructed-week sampling (CWS) was used to draw a sample of the news articles for in-depth analysis from each of the selected country or territory. CWS was used because it enables sampling of news media data to generate themes and frequency of themes representative to the news media data within a timeframe of less than 5 years by addressing cyclic variation in news media contents over the course of a week [20, 21].

### Data handling and analysis

Kruskal Wallis test was used to examine whether daily news counts changed from 2015 to 2018, stratified by media language and country/territory, respectively. If Kruskal Wallis test detects an overall significant difference in daily news counts among the 4 years, post-hoc Dunn’s pairwise comparison with Bonferroni adjustment for *p* values would be run to determine which year has the daily news counts different from other years. In addition, Wilcoxon–Mann–Whitney test was used to compare daily AMR news counts in November and non-November within the same year, stratified by media language and by country/territory, respectively, to inform whether the global WAAW had an effect on media attention for AMR risk or not. To determine main triggers of peak media attention for AMR risk, we first defined the peak day of news media attention for AMR risk as the day with the number of news articles exceeding three times the mean daily number of articles on AMR [22]. News articles on the peak days were examined to identify the most-frequently-reported events which were defined as the key events that drove peak media attention for AMR.

The content coding was conducted for articles chosen using CWS and guided by framing analysis and the mental-models approach (MMA). In communication context, framing is defined as a process to ‘select certain aspects of a perceived reality and make them more salient’ [23]. The aspects selected and the way to make them salient in the news headlines can predominantly shape an audience’s mental representations of ‘problem definition’ (i.e., what is it?) [23]. The MMA is a common approach used to link audience’s mental representations of a health hazard to risk communication [24]. In risk communication, MMA asks questions of what the hazard is, what causes it, what the consequences are, and whether it can be controlled and how. All are important questions to evaluate how communication materials could shape the audience’s mental representation of risk [24].

We first developed a tentative coding scheme focusing on: *AMR frames* (i.e., what is AMR?), *cause* (i.e., what causes AMR?), *social actors* (i.e., whose fault is it or who should be blamed for AMR?), *consequence* (i.e., what are the consequences of AMR?), and *controllability and solution* (i.e., can AMR be controlled and how?), based on analysis of a random subset of 100 eligible news articles. Framing on AMR was analysed based on articles’ headlines, which may substantially impress readers about what AMR is, while other aspects of AMR were analysed based on examination of the full content of the reports. Two research assistants were trained to reliably use the coding scheme to analyse all articles selected using CWS, while maintaining open to allow new codes emerging during coding. The coding scheme was constantly refined during the coding process based on regular discussions among the research team (Appendix Table 7). Inter-rater reliability was assessed by calculating Cohen’s Kappa of which a value of 0.6 or above indicates adequate reliability. To ensure coding accuracy, a random subset of 10% of all codes were further checked by the first author. Any discrepancies in coding were solved by going back to the relevant data and joint discussions among the research team. Each code was compared by year, media language and country/territory using both descriptive analyses and logistic regression models.

## Results

**Fig. 1:**
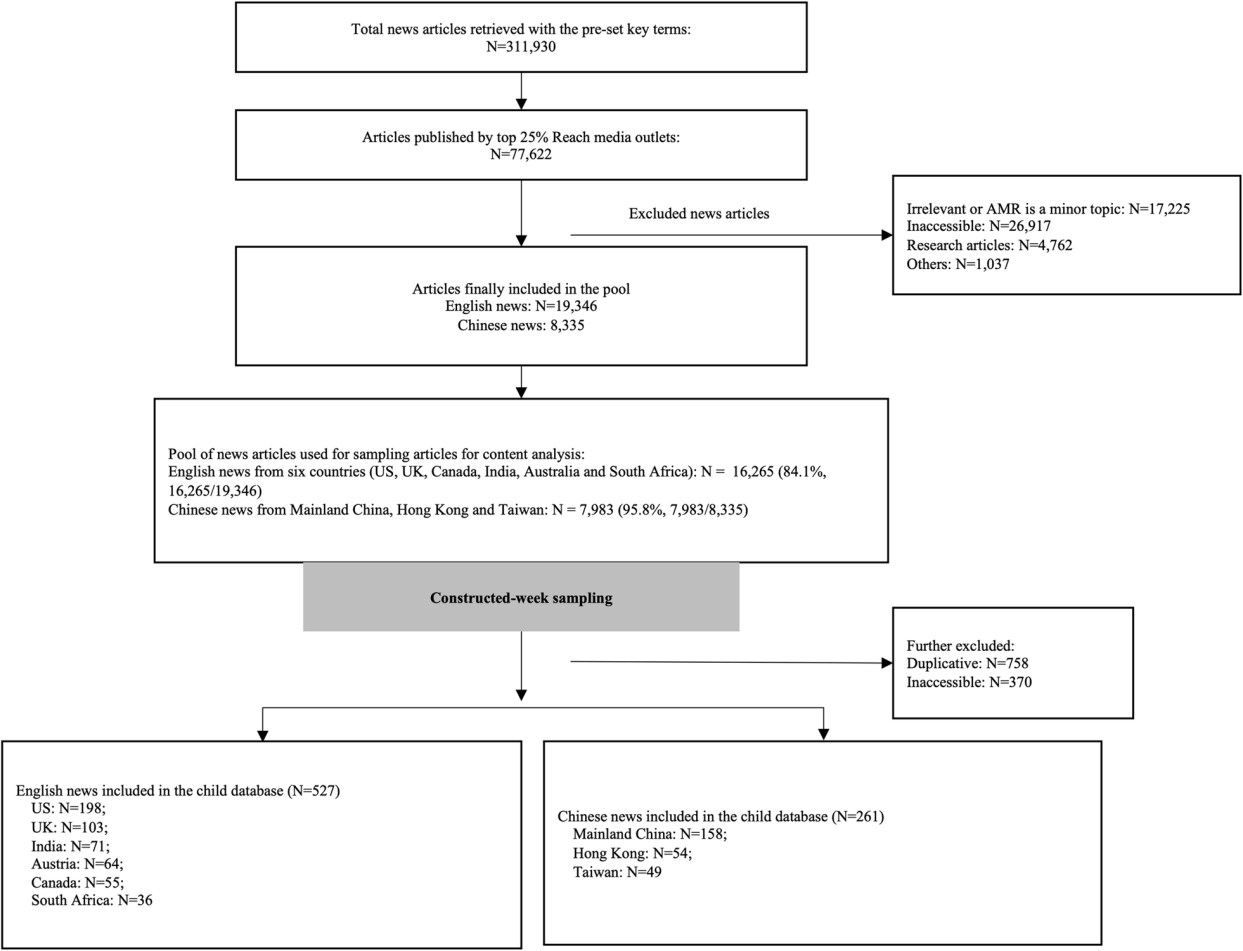
Flow chart of news article selection and screening procedure

A total of 19,346 English news articles and 8335 Chinese news articles were included in the pool for analysing media attention for AMR risk (Fig. 1). The geographic locations of these news articles covered a total of 133 countries in the world. Most English news articles were from the US (45.2%), the UK (18.1%), India (8.7%), Australia (5.1%), Canada (4.9%) and South Africa (2.1%). Most Chinese news articles were from Mainland China (82.7%), Hong Kong (7.4%) and Taiwan (5.6%). A total of 788 news articles from these countries and territories were drawn using CWS for in-depth analysis on the representations of AMR risk (Fig. 1).

### Temporal change of news media attention for AMR risk

Kruskal Wallis test indicated that daily AMR news counts were significantly different among the 4 years in both the English and Chinese media (Table 1). Post-hoc pairwise comparisons indicated that for the English news, daily news counts in 2016 and 2017 but not 2018 were significantly higher than that in 2015, while for the Chinese news, daily news counts significantly increased from 2015 to 2016 and from 2016 to 2017 but maintained relatively stable from 2017 to 2018 (Table 1). The Wilcoxon–Mann–Whitney test also indicated that for the English news, daily news counts were significantly more in November compared to other months (November Effect) in the year of 2016 and 2018; for the Chinese news, the November Effect was detected in 2015, 2016 but not in 2017 and 2018 (Table 1).

**Table 1:** Comparisons of daily AMR news counts by November and non-November months and by year, stratified by media language

Statistics	English	Chinese
Mean daily counts	13.24	5.70
Standard deviation	18.24	8.83
Median daily counts	8	3
Range of daily counts	0–335	0–81
Difference in daily counts. (z-score based on Wilcoxon–Mann–Whitney test) by year in non-Nov. versus:		
Nov. 2015	− 0.26	− 2.49*
Nov. 2016	− 2.02*	− 4.86***
Nov. 2017	− 0.65	− 0.57
Nov. 2018	− 2.19*	− 0.22
Differences in daily counts among years (Kruskal Wallis test)^a^	11.24*	70.84***
Post-hoc Dunn’s pairwise comparison between years (z-score)		
2015 versus 2016	− 2.61*	− 4.53***
2015 versus 2017	− 3.13*	− 7.49***
2015 versus 2018	− 1.88	− 7.05***
2016 versus 2017	− 0.52	− 2.96**
2016 versus 2018	0.73	− 2.53*
2017 versus 2018	1.24	0.44

*p < 0.05; **p < 0.01; ***p < 0.001

^a^Chi-square value of Kruskal Wallis test with three degrees of freedom

By country/territory, daily AMR news counts had increased significantly in Mainland China since 2016, and in India and South Africa since 2017, but increased temporality in Australia in 2016 and 2017 and in Taiwan in 2017 (Table 2). There were no temporal changes in daily AMR news in the US, UK, Canada, and Hong Kong during 2015–2018 (Table 2). In addition, the November Effect was only detected in Australia in 2016, in South Africa in 2015 and 2018, in India in 2016 and 2018, and in Mainland China in 2015 and 2016.

**Table 2:** Comparisons of daily AMR news counts by November and non-November months and by year, stratified by country or territory

Statistics	US	UK	CA	AU	SA	IN	MC	HK	TW
Mean daily counts	6.04	2.40	0.70	0.71	0.27	1.15	4.83	0.47	0.33
Standard deviation	11.56	5.56	1.68	1.49	1.05	1.97	8.09	1.28	1.00
Median daily counts	3	1	0	0	0	0	2	0	0
Range of daily counts	0–236	0–71	0–20	0–23	0–22	0–22	0–73	0–15	0–13
Difference in daily counts. (z-score based on Wilcoxon–Mann–Whitney test) by year in non-Nov. versus									
Nov. 2015	0.38	− 1.80	− 0.98	0.15	− 2.66**	− 1.73	− 2.97**	− 0.60	− 0.90
Nov. 2016	− 1.28	1.00	− 1.14	− 2.65**	0.96	− 2.95**	− 5.17***	− 0.67	1.09
Nov. 2017	0.16	1.11	0.58	− 1.50	0.01	0.05	0.22	− 0.78	− 0.82
Nov. 2018	0.02	− 1.06	− 0.43	0.08	− 4.10***	− 2.22*	− 0.32	0.72	− 1.60
Differences in daily counts among years (Kruskal Wallis test)^a^	10.18*	2.88	2.67	14.88**	12.29**	36.66***	61.79***	7.06	18.42***
Post-hoc Dunn’s pairwise comparison between years (z-score)^b^									
2015 versus 2016	− 1.67	–	–	− 3.74***	− 2.10	0.11	− 4.57***	–	1.32
2015 versus 2017	− 0.31	–	–	− 2.70*	− 2.44*	− 5.06***	− 6.52***	–	− 2.84
2015 versus 2018	1.52	–	–	− 2.12	− 3.40**	− 2.72*	− 7.06***	–	− 0.09
2016 versus 2017	1.35	–	–	1.03	− 0.34	− 5.18***	− 1.95	–	− 4.17***
2016 versus 2018	3.19**	–	–	1.61	− 1.30	− 2.83*	− 2.49*	–	− 1.41
2017 versus 2018	1.84	–	–	0.58	0.96	2.34	− 0.54	–	2.75*

*CA* Canada, *AU* Australia, *SA* South Africa, *IN* India, *MC* Mainland China, *HK* Hong Kong, *TW* Taiwan

*p < 0.05; **p < 0.01; ***p < 0.001

^a^Chi-square value of Kruskal Wallis test with three degrees of freedom

^b^Post-hoc Dunn’s pairwise comparison was only conducted when Kruskal Wallis test indicates that there are significant differences in daily news counts among years

### Main triggers of peak media attention for AMR risk

Table 3 shows that peak media attention for AMR risk was mainly driven by events categorized as ‘official reports assessing AMR risk’, ‘reports of AMR human infections/outbreak’, ‘discovery of new AMR solutions’, ‘discovery of new AMR genes/strains’ and ‘official/organizational actions on tackling AMR’, but seldom by reports of antimicrobial misuse/overuse.

**Table 3:** Categories of events that triggered peak media attention to AMR by media language, 2015–2018

Categories of events that triggered peak media attentions	English media (N = 46)	Chinese media (N = 66)	Both Eng. & Chi. Media (N = 22)
Official reports assessing AMR risk (magnitude and consequence)	11 (23.9%)	10 (15.2%)	5 (22.7%)
Reports of AMR human infections or outbreaks	9 (19.6%)	12 (18.2%)	4 (18.2%)
Reports of new AMR solutions	8 (17.4%)	12 (18.2%)	2 (9.1%)
Discovery of new AMR genes/strains	7 (15.2%)	9 (13.6%)	6 (27.3%)
Official/organizational actions on tackling AMR	4 (8.7%)	11 (16.7%)	2 (9.1%)
Reports of new sources of AMR infections	3 (6.5%)	5 (7.6%)	1 (4.5%)
Reports of antibiotic misuse/overuse	2 (4.3%)	2 (3.0%)	1 (4.5%)
World Antimicrobial Awareness Week	1 (2.2%)	2 (3.0%)	1 (4.5%)
Individual experts’ talks on AMR risk	1 (2.2%)	3 (4.5%)	0 (0.0%)

**Fig. 2:**
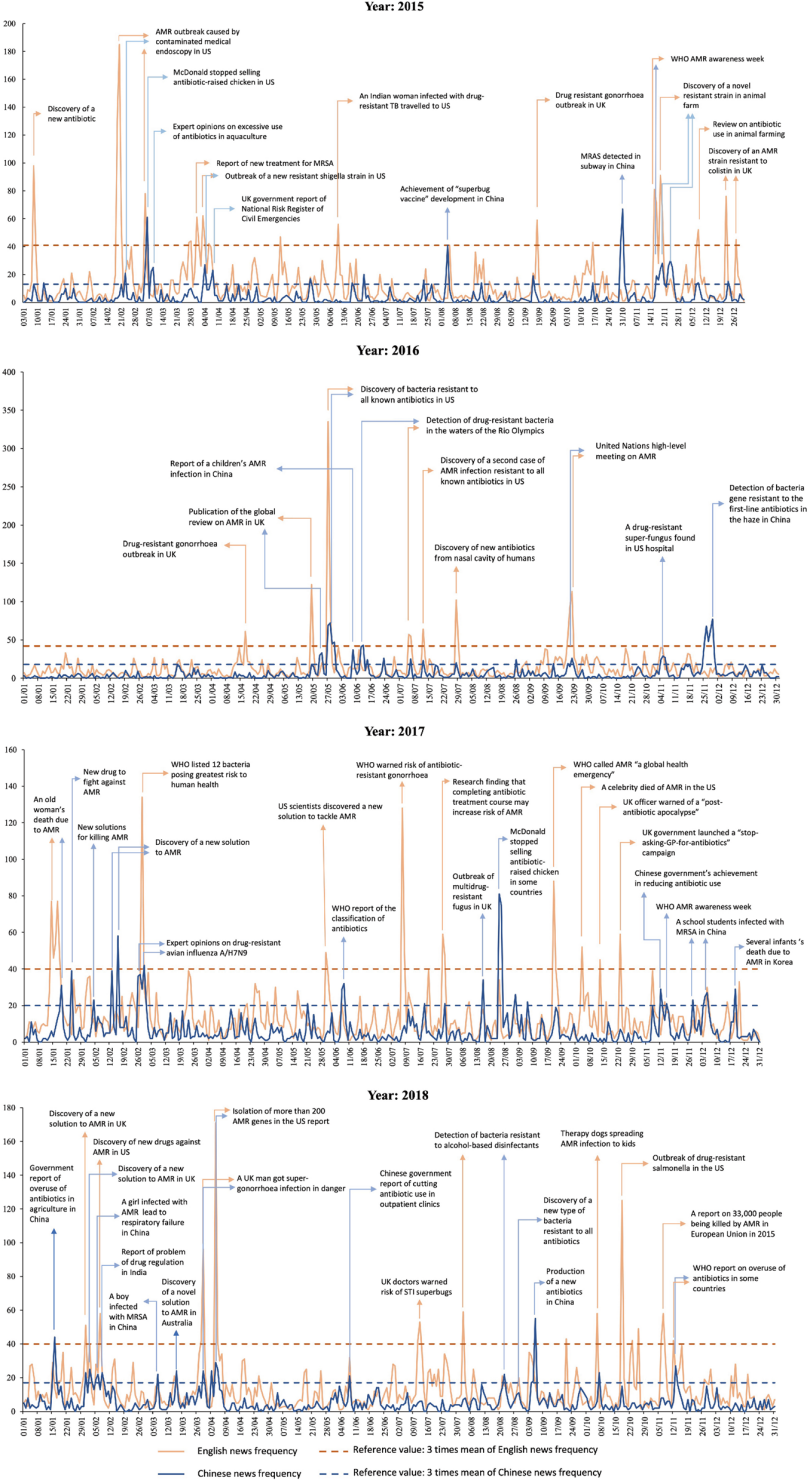
Main events that triggered peak media attention to AMR, 2015–2018

Figure 2 highlights key events that drove media attention for AMR risk by media language and year. It appeared that the media attention for AMR risk tend to peak in response to attributing AMR risk to organizational faults such as “AMR outbreak caused by contaminated medical endoscopy in the US” for English news and “McDonald stopping selling antibiotic-raised chicken (in other places but not China)” for Chinese news. Other key events that drove media attention were the research discovery of new AMR risk or solutions such as “discovery of bacteria resistant to all known antibiotic” and “isolation of more than 200 AMR genes” in the US, and “MRSA detected in subway”, “detection of bacteria gene resistant to the first-line antibiotics in the haze” and “production of a new antibiotics” in China. The World Health Organization (WHO) played an active role in driving media attention to the issues of AMR in 2017 (e.g., “report of 12 bacteria posing AMR risk to human health”, “warning of risk of antibiotic-resistant gonorrhoea” and “framing AMR as a global health emergency”). Although the WAAW was identified to be a trigger for a major attention peak in both English and Chinese news in 2015, there was only a slight increase in mentioning WAAW in media in November of 2017 and 2018, respectively.

### Representations of AMR risk

#### Headline framing of AMR

**Fig. 3:**
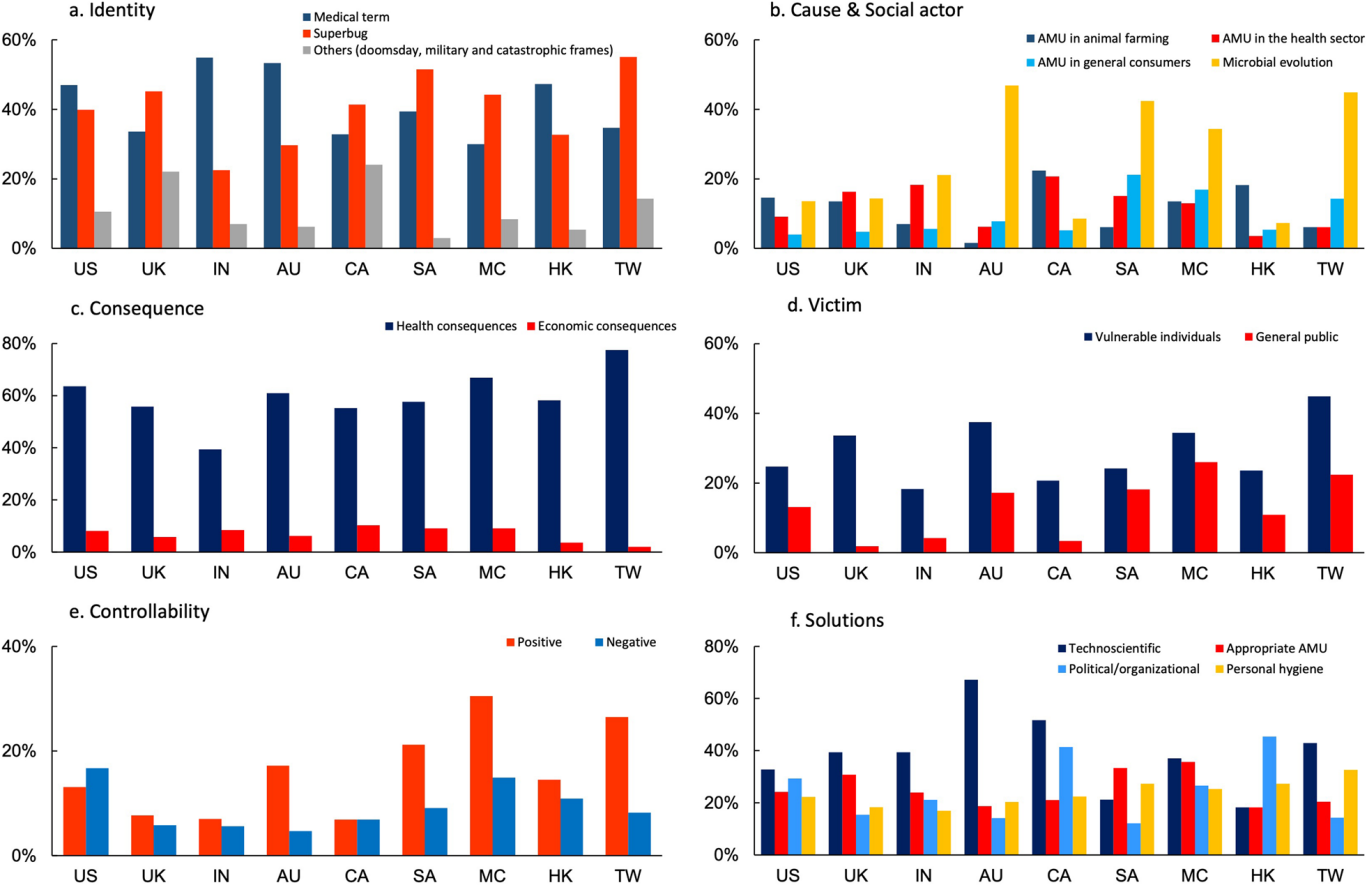
Proportions of the respective representations of AMR risk by country or territory, 2015–2018. *AMU* antimicrobials use, *US* The United States, *UK* The United Kingdom, *IN* India, *AU* Australia, *CA* Canada, *SA* South Africa, *MC* Mainland China, *HK* Hong Kong, *TW* Taiwan

Medical terms were the most frequently used (41.0%, 323/788) including general medical terms (e.g., “antibiotic resistance” or “bacterial resistance”) and drug-specific (e.g., colistin-resistant) or pathogen-specific medical terms (e.g., MRSA), followed by the ‘superbug’ frame (40.0%, 315/788) (Table 4). The ‘doomsday’ frame (e.g., “antibiotic apocalypse” and “post-antibiotic era”), ‘military’ frame (e.g., framing ARM as a “battle” or “war”) and ‘catastrophic’ frame (e.g., crisis) in the news headlines were less common (Table 4). While India, Australia, Hong Kong and the US tend to use the medical terms, Taiwan, South Africa, UK and Mainland China tend to use the ‘superbug’ frame (Fig. 3a).

**Table 4:** Frequency of content codes across media language based on the analysis of the 788 news articles selected using constructed-week sampling

	English news (N = 527) (%)	Chinese news (N = 261) (%)	Total (N = 788) (%)
*Headline framing on AMR*	84.2	78.9	82.5
Medical terms	44.2**	34.5	41.0
Superbug	38.3	43.3	40.0
Doomsday (e.g., antibiotic apocalypse, post-antibiotic era)	4.7	6.5	5.3
Military term (e.g., battle, war)	6.3*	2.3	4.9
Catastrophic (e.g., crisis, disaster)	1.5	1.1	1.4
*Cause*	49.5***	63.2	54.1
Inappropriate AMU	33.8*	42.5	36.7
AMU in animals	11.9	15.3	13.1
AMU in the health sector	12.9	10.0	11.9
AMU in the general consumers	5.9***	14.2	8.6
Microbial evolution	19.9**	30.6	23.5
*Consequence*	58.6*	67.0	61.4
Health consequences	57.1**	67.0	60.4
Economic consequences	7.6	6.9	7.4
*Victim*	35.1***	52.1	40.7
Vulnerable individuals	26.6*	34.5	29.2
General public	9.5***	21.8	13.4
*Controllability*	21.2***	39.5	27.3
Positive	11.4***	26.4	16.4
Negative	9.8	13.0	10.9
*Solution*	78.9	81.2	79.7
Technoscientific solutions	40.4	34.1	38.3
Appropriate antimicrobial use (AMU)	26.0	29.1	27.0
Political/organizational solutions	23.5	28.7	25.2
Personal hygiene	21.1	26.8	23.0
Others (e.g. vaccination, breastfeeding)	4.5	2.7	3.9

*p < 0.05; **p < 0.01; ***p < 0.001

All *p* values were calculated based on Pearson chi-square differences in the frequency of the code between the English and Chinese new articles

#### Causes and social actors

Of the 788 articles, 426 (54.1%) communicated the causes of AMR, which was more common in Chinese news (χ^2^_(1)_ = 13.18, *p* < 0.001). Two important causes—inappropriate AMU (36.7%, 289/788) and microbial evolution as a process of AMR (23.5%, 185/788) were identified. Communicating microbial evolution as a process of AMR was more common in 2017–2018 than in 2015–2016 (χ^2^_(3)_ = 16.82, *p* = 0.001), but when microbial evolution was communicated, only 25.9% (48/185) mentioned human behaviours of inappropriate AMU as a cause to accelerate the AMR process. When communicating inappropriate AMU as a cause, most referred this to inappropriate AMU in animals for which farmers, food companies and veterinarians were blamed (13.1%, 103/788), followed by inappropriate AMU in the health sector for which patients, doctors and hospitals were blamed (11.9%, 94/788) while only 8.6% (68/788) blamed inappropriate AMU among general consumers (Table 4). Australia, Taiwan, South Africa, and Mainland China most frequently communicated AMR as a process of microbial evolution (Fig. 3b). India emphasized inappropriate AMU in the health sector more than in animal farming or in the general public; Australia and South Africa emphasized that in the health sector and general consumers more than that in animal husbandry, while the US, UK and Canada emphasized this more in animal farming and the health sector more than that in general consumers (Fig. 3b).

#### Consequences and victims

Around 60% (61.4%, 484/788) communicated the consequences of AMR, which was more common in Chinese news (χ^2^_(1)_ = 5.22, *p* = 0.022). Most communicated the health consequences of AMR (60.4%, 476/788) while 7.4% (58/788) communicated the economic consequences (Table 4). The frequency of communicating the health and economic consequences of AMR was comparable across countries/territories apart from India which less frequently communicated the health consequence of AMR (Fig. 3c).

The *victim* (for whom AMR risk is relevant) emerged as a new theme in the analysis of consequences of AMR. Vulnerable individuals (29.2%, 230/788) were more commonly mentioned as the victim of AMR than the general public (Table 4). Communicating the general public as the victims of AMR increased by year (9.2% in 2015, 11.3% in 2016, 15.4% in 2017 and 17.8% in 2018, χ^2^_(3)_ = 7.36, *p* = 0.0061). Mainland China, Taiwan, South Africa, Australia and the US were more likely to communicate the general public as the victims of AMR than other sites (Fig. 3d).

#### Controllability and solutions

Of the 788 news articles, 129 (16.4%) used a positive tone such as “successfully harness”, “17”, and “hope” (of new solutions) while 86 (10.9%) used a negative or pessimistic tone for the controllability of AMR (e.g., “fail” (to treat), “untreatable”, “difficult to control” and “uncontrollable”). While Mainland China, Taiwan, South Africa and Australia tend to use a positive tone for controllability, the US tend to use a negative tone for controllability (Fig. 3e).

*Solutions* were the most frequently communicated aspects of AMR risk, of which, technoscientific solutions (38.3%, 302/788) (e.g., discovery of new antibiotics, new treatments or other technical solutions) were most common, followed by appropriate AMU (27.0%, 213/788) (mostly in health sector and animal husbandry), and political/organizational solutions (25.2%, 199/788) (e.g., policies to regulate AMU in humans and animals, and strengthening surveillance of AMR and infection control). Around 23.0% (181/788) mentioned personal hygiene (e.g., frequent handwashing and avoiding close contact with animals) as the solution and several mentioned vaccinations (3.5%, 28/788). There was an increase in frequency of communicating technoscientific solutions by year (χ^2^_(3)_ = 28.88, *p* < 0.001). Australia, Canada and Taiwan were the three sites that most frequently reported the technoscientific solutions, while Hong Kong, Canada and the US more frequently reported the political and organizational solutions, and Mainland China, South Africa and UK more frequently reported appropriate AMU as the solutions compared with other sites (Fig. 3f).

## Discussion

With an unprecedented global attention to AMR at political levels since 2015 [25–28], an overall increase in media attention given to AMR issues was identified from 2015 to 2016 and 2017 in both English and Chinese news media. However, media attention for AMR appeared to decline from 2017 to 2018 though the change was not significant. By country/territory, we found that the increase in media attention for AMR from 2015 to 2016 and/or 2017 mainly happened in less developed countries (South Africa and India) and territories (Mainland China and Taiwan) but not in more developed countries (US, UK and Canada) and territory (Hong Kong). This may be due to the already relatively high baseline media attention for AMR in those areas (e.g., US and UK) or the insufficient development or implementation of their AMR action strategies. For less developed countries and territories, the temporary increase in media attention for AMR appeared to be not sustained in later years. Overall, it indicates that sustained media attention for AMR may be challenging when the ‘news’ value of AMR declines.

AMR outbreaks or risk attributing to organizations, scientific discovery of new AMR risk or solutions, and official assessment reports of AMR risk remained the most important drivers of peak media attention for AMR. While research on inappropriate AMU was seldom identified as the drivers of peak media attention despite a substantial increase in relevant research over the past decade. Such patterns of media response tend to centralize the role of government, organizations and scientists while marginalizing the role of general individuals in tackling AMR. The events that triggered peak media attention to AMR (Fig. 2) indicate that WHO, healthcare professional and scientists are the major social actors while other sectors such as animal health and food industry remained disengaged in communicating about AMR risk, indicating insufficient multisectoral effort in communicating AMR. In China, several peaks of media attention for AMR were triggered by extreme cases of children infected with AMR (Fig. 2). Such news can be alarming for the public but tend to shift public attention for a medical solution for AMR.

Overall, WAAW was only identified as a trigger of peak media attention for AMR risk in 2015 and a slight increase in media attention for AMR risk in 2017 and 2018, respectively. In our analyses stratified by country/territory on the November Effect, we found that increases in media attention for AMR in November versus other months mainly detected in less developed countries and territory and such increases were not consistently identified in November of 2015–2018. This indicates that WAAW was insufficiently implemented in the countries and territories included in our study. The WHO was found to play a crucial role in promoting media attention for AMR risk in some years and in reorienting media attention to the centrality of human behaviours in the issues of AMR [30, 31]. However, without strategic planning in the design and implementation of the global communication campaigns on AMR [32] and the commitment of individual countries, WHO’s effort alone may not generate enough sustained effect in promoting public awareness of AMR [29].

Medical terms were the most common frame in headlines of AMR news, which appears more accurate and informative and is usually favoured by scientists, official organizations and health professionals. However, the heterogenous medical terms used can be a source of misunderstanding and barrier for public understanding and engagement with AMR [33]. In addition, the medical frames can drive primarily medical approach to AMR and thereby place the burden of tackling AMR to health sectors and health professionals. The ‘superbug’ frame can vividly describe a human ‘enemy’ that is ‘clever’ and ‘powerful’, resisting all/most antimicrobials, and was commonly used in the headlines of AMR news. Its simplistic expression can be easily used by the general public in their daily communication but may obscure the mechanism and function of AMR and hence lead to public misperceptions [34]. Although using the ‘superbug’, ‘doomsday’, ‘military’ and ‘catastrophic’ frames to depict AMR risk may easily evoke feelings of anxiety and a sense of urgency about AMR, they likely put the drug-resistant microorganisms into the position of an outside ‘enemy’ and tend to set aside human responsibility in the agenda of AMR [7, 34]. It is recommended that a common medical term that can be easily understood by the general public, such as “drug-resistant infections”, should be used in the media to improve public understanding and engagement with the issues of AMR [33].

There was an increasing trend of communicating the biological process of AMR from 2015–2016 to 2017–2018 in news media, which may facilitate public understanding about the function of AMR as a process of microbial evolution rather than the sudden emergence of ‘superbugs’ [34, 35]. However, while microbial evolution was communicated as a process of AMR, the human behaviours of inappropriate AMU that accelerated this process was incompletely communicated, attenuating human responsibility in the issues of AMR. In addition, the news remained focused on blaming the social actors of animal husbandry and the health sectors in AMU. The role of general consumers, who fundamentally drive consumption of antimicrobials and shape the animal-farming and animal-production systems, was generally downplayed. It is also striking to notice that the inappropriate AMU in animal sectors remained downplayed also in the process of AMR in countries, such as China, South Africa, and India, that are major countries of antibiotic use in animal husbandry [36, 37]. This may reflect the weaknesses of the AMR surveillance systems for animal sectors (e.g., insufficient funding and lacking standardized guideline for AMR testing) in these countries and the insufficient implementation of the One Health approach in their national action plans [38–40]. The lack of media coverage in AMU in animal sectors may in turn hinder public support for the policy regulation on AMU in farming and production of food animals.

Communication about the consequences of AMR mainly focused on the health consequences for vulnerable groups/individuals, driving actions for AMR to be primary a healthcare issue with hospitals, doctors and patients being positioned as the central agency of AMR. The multifaceted consequences of AMR, including its detrimental effects on the healthcare system, social development, economic and food security were seldom communicated, hindering the implementation of the one-health approach and intersectoral collaboration for tackling AMR [41]. Despite all this, a growing trend of emphasizing the general public as the victim of AMR, particularly in Mainland China, Taiwan, South Africa, Australia and the US, may indicate an increasing awareness of AMR as a global threat for which collective actions are important.

Although solutions of AMR were most commonly communicated, technoscientific solutions were the main theme of AMR solutions, which showed an increasing trend over time by year. The tendency to emphasize the role of science and medicine in tackling AMR will continue to drive resource allocation to increase investment in the development of new antibiotics, for instance, rather than in public health to change human behaviours of AMU, personal hygiene and vaccination. The issue of AMR was seldom framed as being controllable or actionable. In the US news, while consequences of AMR were a dominant theme, AMR tend to be framed as something that was difficult to control. Such patterns of media representations could shape pessimistic public responses to the risk of AMR. While emphasizing the risk of AMR (e.g., consequences) is important to raise public awareness, it is equivalently important to frame AMR as something controllable and actionable to empower the public in taking actions on it.

Our study has several limitations. Firstly, our analysis only included English and Chinese news due to resources restriction. The English news merely included six countries for which English was the official language or one of the main official languages. For countries with multiple official and other native languages such as India and South Africa, our findings may reflect only the partial picture of media representations of AMR risk in these countries. It is possible that our results may be subject to biases if representations of AMR risk differ by English and non-English news media in these countries. Secondly, the news articles were retrospectively retrieved and thus access to some news articles was lost over time. Thirdly, merely the textual contents of online news articles were analysed and thereby some of the nuances surrounding the understanding about representations of AMR news by print media, social media and other forms of media data were lost. Furthermore, while our study focused on how AMR risk was communicated in news media to infer their influences on public mental representations of AMR risk, future studies are needed to test how different representations of AMR risk affects public mental representation of AMR risk.

To conclude, an overall increasing trend in media attention given to AMR issues was identified from 2015 to 2016–2017 but media attention for AMR risk appeared to decline in 2018. The representations of AMR risk in English and Chinese news media in 2015–2018 can shape public perceptions of the centrality of the roles of scientists, government and commercial organizations, rather than general consumers in the agenda of AMR. Our study also indicates the insufficient implementation of the One Health approach and intersectoral collaboration in AMR surveillance, impact assessment and communication across countries. The implementation of the One Health concept in tackling AMR requires intersectoral engagement and collaboration. First, the WHO should play a crucial role in helping countries particularly low-resources countries for establishing the AMR surveillance systems not only for the health sector but also for the animal and environment sectors. This may require both funding and technical support with the coordination of the WHO. Second, individual countries should establish or refine their national action plans including strengthening surveillance of AMR and AMU in animal and environment sections and establish a platform for sharing AMR (i.e., AMR epidemiology and the multifaced consequences of AMR) and AMU data across sectors. AMR and AMU data can also be shared with the media to facilitate AMR risk communication. Local media should reflect their communication strategies about AMR risk upon the One Health principle, local AMR and AMU data, and the global media discourse and strategies in the issue of AMR to urge policy decision making and reshape research direction in local countries. The communication should reflect the multifaceted consequences of AMR, responsibilities of multi-level sectors rather than one single sector, and the controllability and actionable measures to facilitate engagement of multi-sectors and collective actions for tackling AMR.

## Data Availability

The datasets generated during and/or analysed during the current study are available from the corresponding author on reasonable request.

## Appendix

See Tables 5, 6 and 7.

**Table 5:** Searching key words for Chinese and English news

#1 Headlines contain:	Drug-resistan^*^; multidrug-resistan^*^; post-antibiotic^*^; MDR-TB; carbapenmen-resistan^*^; artemisinin-resistan^*^; superbug^*^; resistant-micro^*^; methicillin-resistan^*^; vancomycin-resistan^*^; colistin-resistan^*^; fluoroquinolone-resistan^*^; cephalosporin-resistan^*^; penicillin-resistan^*^; drug resistance; antibiotic resistance; antimicrobial resistance
#2 Contents contain:	Drug-resistan^*^; multidrug-resistan^*^; post-antibiotic^*^; MDR-TB; carbapenmen-resistan^*^; artemisinin-resistan^*^; superbug^*^; resistant-micro^*^; methicillin-resistan^*^; vancomycin-resistan^*^; colistin-resistan^*^; fluoroquinolone-resistan^*^; cephalosporin-resistan^*^; penicillin-resistan^*^; drug resistance; antibiotic resistance; antimicrobial resistance
#3 Headlines contain:	Bacteria^*^; virus; viruses; viral; microbial; microbe; microbes; microorganism^*^; protozoa^*^; parasite^*^; fungal; fungi; clostridium difficile; Staphlylococcus aureus
#4 Headlines contain:	Antibiotic^*^; antivir^*^; antiretroviral; antifungal^*^; antimalarial^*^; antiprotozoal^*^; anthelmintic; antimicrobial^*^; anti-micro^*^; multi-drug; carbapenmem; methicillin; vancomycin; oxacillin; fluoroquinolone^*^; cephalosporin^*^; colistin; penicillin^*^
#5 Headlines contain:	Pneumoniae; gonorrhoea; E. coli; tuberculosis; malaria; MRSA; ART

Article searching strategies were: #1 OR (#2 AND (#3 OR #4 OR #5))

**Table 6:** Inclusion and exclusion criteria for screening the eligibility of the news articles

	Details of the criteria
Inclusion criteria	News articles reported any of aspects of AMR risk: what AMR is, who or what causes it, what the consequence is, whose responsibility, whether AMR can be controlled and how
Exclusion criteria	1. Full texts were not public-accessible
	2. AMR is mentioned as a minor topic rather than the main topic in the article
	3. Research articles or project reports for which the target audience are professional rather than the general public
	4. Advertisements e.g., new drugs, conference advertisements, advertisement for recruiting subjects for an AMR study
	5. Articles is about other drug resistance (e.g., cancer drug resistance)

**Table 7:** Coding scheme for content analysis on the representations of AMR risk

	Description
*Headline framing on AMR*	How AMR or AMR risk was labelled or interpreted in the headlines
Medical terms	The general medical terms such as antibiotic resistance, drug-resistant bacteria, or more specific medical term such as MSAR, multidrug-resistant *Acinetobacter* baumannii were used
Superbug	Superbug, superbugs or bacteria- or disease-specific superbugs such as super-gonorrhea was used to labelled AMR
Doomsday	The way AMR risk was labelled aimed to give alarming that AMR risk could lead to antibiotic apocalypse or post-antibiotic era when antibiotic of last resort became ineffective
Military term	AMR risk was labelled as a battle or war for which weapons were needed
Catastrophic	AMR risk was labelled as a crisis, disaster, global threat or something that was out of control
*Cause and social actors*	What cause AMR and who/what should be blamed for the problem of AMR
Misuse or overuse of antimicrobials	Misuse or overuse of antimicrobials particularly antibiotics including misuse or overuse of antimicrobials. The social actors for these can be sectors/individuals who were responsible for animal farming or health care, and the general consumers
Microbial evolution	The change of microbials themselves as a consequence of natural evolution was mentioned as the cause of AMR
*Consequence*	The consequences caused by AMR
Health consequences	Health consequences in humans due to AMR. This included sickness, infections becoming difficult to treat and death
Economic consequences	The economic loss due to AMR
Victims	Who was affected by AMR (whose risk is relevant). Two subcategories were found for this including vulnerable groups/individuals (patients, children or older people) or the general public (e.g. AMR affects AMR)
*Controllability*	Whether AMR can be controlled or not
Positive	The articles used a positive tone about the controllability of AMR such as words of “successfully harness”, “turning point”, and “hope” (of new solutions)
Negative	The articles presented a pessimistic tone about the controllability of AMR using words such as “fail” to treat, “untreatable”, “difficult to control” and “uncontrollable”
*Solution*	
Technoscientific solutions	This includes discovery of new antibiotics, new treatments for resistant bacterial infections, and new technologies such as those that can be used to detect and kill drug-resistant bacteria in the environment
Appropriate antimicrobial use (AMU)	This emphasizes the importance of appropriate use of antimicrobials in humans and animals. It also includes those mentioned the importance of health education to raise people’s awareness of AMR risk and change their behaviours of antibiotic use
Political/organizational solutions	This emphasizes the responsibility of government or organization in the control of AMR. Solutions can be making policies to regulate antimicrobial use in humans and animals, strengthening surveillance of AMR and infection control, increasing funding for AMR research or control of AMR, or establishing new organizations for the control of AMR
Personal hygiene	This includes those emphasizing the importance of personal hygiene behaviours such as frequent handwashing, disinfection and avoiding close contact with animals
Others (e.g. vaccination, breastfeeding)	Other measures not included in the above such as vaccination and breastfeeding to promote personal immunity

## Abbreviations

AMR: Antimicrobial resistance
AMU: Inappropriate antimicrobial use
WAAW: World antimicrobial awareness week
CWS: Constructed-week sampling
US: United States
UK: United Kingdom
MMA: Mental-models approach
WHO: The World Health Organization
